# Is Negative Pressure Wound Therapy with Instillation Suitable for the Treatment of Acute Periprosthetic Hip Joint Infection?

**DOI:** 10.3390/jcm10153246

**Published:** 2021-07-23

**Authors:** Nicholas A. Beckmann, Maximilian G. Hanslmeier, Georg W. Omlor, Manuel Feisst, Michael W. Maier, Burkhard Lehner

**Affiliations:** 1 Department of Orthopaedic and Trauma Surgery, University Hospital Heidelberg, Schlierbacher Landstrasse 200A, 69118 Heidelberg, Germany; Maximilian.Hanslmeier@med.uni-heidelberg.de (M.G.H.); Georg.Omlor@med.uni-heidelberg.de (G.W.O.); burkhard.lehner@med.uni-heidelberg.de (B.L.); 2 Institute of Medical Biometrics and Informatics, University Hospital Heidelberg, Im Neuenheimer Feld 130.3, 69120 Heidelberg, Germany; feisst@imbi.uni-heidelberg.de; 3 Swabian Joint Center, Christoph Strasse 7, 70178 Stuttgart, Germany; maier@sgz-stuttgart.de

**Keywords:** hip, joint infection, prosthesis, arthroplasty, DAIR, debridement and implant retention, NPWTI, THA, negative pressure wound therapy

## Abstract

Background: Periprosthetic joint infection (PJI) can be devastating for the patient and demanding for the surgeon. In acute PJI, attempts are made to retain the prosthesis by debridement of the infected tissue, targeted antibiotic therapy and an exchange of modular components with implant retention (DAIR). There has been sparse research with adjunctive negative pressure wound treatment with wound irrigation (NPWTI) on the treatment outcome. Questions/purposes: The goal was to assess the efficacy of our protocol of DAIR with adjunctive NPWTI in acute PJI and to reduce the need for later additional DAIR and Irrigation and Debridement (I and D). Patients and Methods: Our cohort of 30 patients (31 hips) with acute PJI was divided into two groups based on symptom presentation up to 6 weeks or >6 weeks from prior (index) surgery (acute early or acute late groups, respectively). All received DAIR with an exchange of modular components and NPWTI with polyhexanide instillation, with the goal of bacterial elimination and biofilm elimination. Postoperatively, the patients were followed up clinically and radiographically for a mean of 4.3 years. Results: Of the 31 PJI hips, 19 were early acute and 12 were late acute. In total, 21 hips had no evidence of residual infection, 10 required further surgical revision: 1 due to dislocation and 9 due to infection. Of these nine, seven had a removal of all the components and two were treated with irrigation and debridement (I and D), with the demise of one patient from pneumonia shortly after the procedure. The Kaplan–Meier 60-month revision free implant survival from infection was 73.2% (CI: 58.9–91.0%) and at the final follow up, the mean Harris Hip Score (HHS) was 81.1 ± 11.8 and the mean WOMAC score was 33.3 ± 20.1. Conclusions: Our results are in line with those reported in prior studies. However, the utility of our protocol is inconclusive and needs further evaluation based on our small cohort and the lack of a control group. Level of Evidence: IV.

## 1. Introduction

Periprosthetic joint infection (PJI) is an infrequent, but devastating complication in 2–3% of joint replacement surgeries and is the third leading cause of re-revision procedures [1,2]. In the USA, the number of infected knee and hip arthroplasties is expected to rise from 18,000 in 2011 to >42,000 in 2020 [1,3,4,5,6] with increases in the frequency of atypical and resistant pathogens [2,7], patient morbidity and mortality and financial cost to the health care system. PJI revision has been reported to have a five-fold mortality rate compared to cases of aseptic revision [8].

The diagnostic criteria and treatment modalities of acute PJI have been the subject of much debate [9,10,11,12]. Parvizi et al. in 2018 conducted a large retrospective review of THA and TKA cases and validated the evidence based diagnostic criteria [10]. However, cases remain in which a diagnosis based on the listed criteria remains uncertain, and clinical judgement remains the final arbiter [10].

Multidisciplinary treatment involves antibiotic therapy and a variety of surgical options including DAIR, one or two stage component exchange and resection arthroplasty with or without arthrodesis. Long-term antibiotic suppression may be an alternative or adjunctive option in select cases [13].

Debridement, antibiotics and implant retention (DAIR), usually combined with modular component exchange, has been the treatment of choice for acute PJI of the hip. However, the definition of acute PJI has been confusing, variable and subject to much debate [9]. Early acute PJI implies an onset of acute symptoms varying from 4 weeks to 3 months after index surgery [3,14,15,16,17,18]. Some authors defined delayed acute PJI [19] as an onset of symptoms 6 or more weeks after index surgery. Acute hematogenous PJI has referred to an acute symptom onset occurring from 4 weeks to more than 2 years after index surgery in a previously asymptomatic hip [20,21].

Our protocol differed from most by our use of repeat DAIR and NPWTI with polyhexanide instillation to target residual bacteria and biofilm in cases with continued positive tissue cultures. The goal of our study was to assess the effectiveness of our protocol for the treatment of acute PJI and to compare our results to others in the literature.

## 2. Materials and Methods

The study was approved by the local ethics committee. All patients provided informed consent for this study in compliance with the 1964 Declaration of Helsinki. 

Our cohort of 30 patients (31 hips) developed acute PJI following a primary or revision THA between 5 November 2005 and 23 December 2013. All were treated at the local university hospital according to the hospital protocol. The patients were followed clinically and radiographically. None were lost-to-follow-up.

We arbitrarily divided our cohort into the following 2 groups of acute PJI based upon the time interval of symptom presentation from the index surgery: 0–6 weeks (early acute-group 1) and >6 weeks (late acute-group 2). Our late acute group incorporated those identified elsewhere as acute hematogenous, since we were unable to identify a primary site of infection in most cases. Their treatment followed our same protocol [9].

Our study had the following 3 main end points: (1) the assessment of surgical success, defined as the absence of detectable infection and no further surgical intervention for infection; (2) the assessment of implant survival, defined as the lack of implant exchange (cup and/or femoral stem) necessitated by infection (infection related implant survival); (3) implant survival from all causes.

### 2.1. Cohort

All received the DAIR procedure with modular component exchange and NPWTI with antiseptic polyhexanide instillation.

The average age at the time of surgery was 64.5 ± 12.7 (range 39–87) years, 20 (67%) were male and 10 (33%) were female patients, 13 (43%) were right and 16 (53%) were left hips. One female patient (3%) had both hips treated for infection. The mean BMI was 27.3 ± 4.5 (range 18.9–39.3) kg/m^2^. Retrospective chart review revealed the following ASA (American Society of Anesthesiologists) classification groupings [22]: Class I: 1 patient (3.3%), Class II: 12 patients (40%), Class III: 16 patients (53.3%) and Class IV: 1 patient (3.3%). Cases with preoperative radiologic evidence of (aseptic) loosening of the cup and/or stem or evidence of current dislocation were excluded. The mean study follow-up period was 4.3 ± 3.7 (range 0–12.3) years.

The elective surgery immediately preceding the development of infection was defined as the index operation and was a primary THA (PTHA) in 21 hips and a revision THA (RTHA) for causes other than infection in 10 hips. The reasons for the index operation are listed in Table 1.

All patients had routine preoperative radiologic studies to exclude stem or cup loosening, lab studies including erythrocyte and white blood cell counts with differential, CRP and additional studies when indicated (CT and/or ultrasound). Preoperative joint aspiration was performed on 17 hips and specimens were submitted for histologic examination and culture. Leukocytosis, elevated CRP levels and positive cultures from exudate and joint aspirate were considered evidence of acute infection according to recent guidelines [3] in addition to clinical findings of redness, swelling, pain, exudate and purulence. No patient had a sinus tract.

Microbial cultures were positive in 27 hips. Four hips were culture negative but had clinical and/or histologic evidence of acute infection that warranted treatment and inclusion in the study.

Patients referred by their outpatient physician received operative treatment the same day if medically stable. Cases with sepsis were initially started on immediate preoperative antibiotic therapy. Perioperatively, patients received thrombotic prophylaxis and after tissue sampling, I.V. Cefuroxime.

All surgeries were performed by surgeons with specialty board certification and several years of additional experience in hip surgery. The operative approach varied but utilized the prior incisional site. Implant stability was confirmed intraoperatively, the joint was dislocated, and modular components were removed to allow improved access for debridement. Necrotic tissue was debrided and 4–6 periprosthetic tissue samples were taken for culture and histology from areas of apparent infection, including the pseudocapsule, interface and synovium. The operative site was irrigated, and modular components were manually disinfected with polyhexanide (Lavanid R 2, Serag-Wiessner & Co. AG, Naila, Germany) and reimplanted to allow patient mobilization. Sonication was not used. PVA (Polyvinylalcohol foam, KCL, Medizinprodukte GmbH, Wiesbaden, Germany) sponges were placed around the endoprosthesis, exiting through the wound, and the vacuum instillation system (KCL Medizinprodukte GmbH, Wiesbaden, Germany) was installed. Timed instillation periods with a 0.04% polyhexanide solution alternated with periods of vacuum suction for 4–6 days. If tissue samples from the most recent debridement were culture positive, the entire process was repeated, including debridement, tissue sampling, modular component removal with disinfection and replacement followed by reinstallation of the vacuum system. This sequence was repeated until the most recent tissue samples became culture negative, consistent with no residual infection [8,23,24,25]. Then, modular components were exchanged for new components and the wound was closed.

All 31 new liners utilized the original locking mechanism.

After pathogen identification, focused antimicrobial therapy was initiated based on the antibiogram and specialist consultation and continued after the termination of DAIR and NPWTI. Intravenous antibiotics were administered for 2 weeks then 6–8 weeks orally, dependent on clinical assessment and laboratory values. No patients received chronic antibiotic suppression. Most patients received a Rifampicin plus Cefuroxime combination and additional antibiotics as indicated by the antibiogram. Radiographs and laboratory values were performed when indicated and the patient was discharged when clinically stable with a dry wound, normal or low and decreasing CRP level and leukocyte count. Patients returned for follow-up assessment at 6 weeks and 6 months and were seen at least yearly in the outpatient clinic and/or by their outpatient physician who provided additional information at the final follow-up assessment.

Hip function was assessed with the following scales at the final follow-up:

Harris Hip Score (HHS) [26] for functional level (scale 0–100; <70 = poor, 70–79 = satisfactory, 80–89 = good, 90–100 = excellent); pain score (scale 0–10; 0 = no pain); WOMAC scale [27] for pain, stiffness and physical ability (scale 0–96; 0 = best); satisfaction score (scale 1–5; 1 = best); UCLA Activity score [28] for physical ability (scale 1–10; 10 = best); Tegner Activity score [29] (scale 0–10; 5 = recreational, 10 = elite competitive sport).

### 2.2. Statistics

Categorical variables were described by absolute and relative frequencies. Postoperative scores were described using mean, standard deviation and range or with median, interquartile range and range, as appropriate.

To estimate the long-term survival for infection-related implant survival, we used a Kaplan–Maier analysis as well as a competing risk analysis. The competing risk was death and was used due to the patient comorbidity and age.

All analyses were performed with the software R Version > 4.0.0 (R Foundation for Statistical Computing, Vienna, Austria) [30].

## 3. Results

### 3.1. Summary of Outcome

All 31 hips received DAIR with a modular component exchange and NPWTI. None were lost to follow up. The mean number of DAIR was 2.6, the median number was 3 (range 1–4) and the Interquartile Range (IQR) was 1. Four patients had one DAIR and NPWTI followed by modular exchange and wound closure after the initial culture proved negative. 

**Figure 1:**
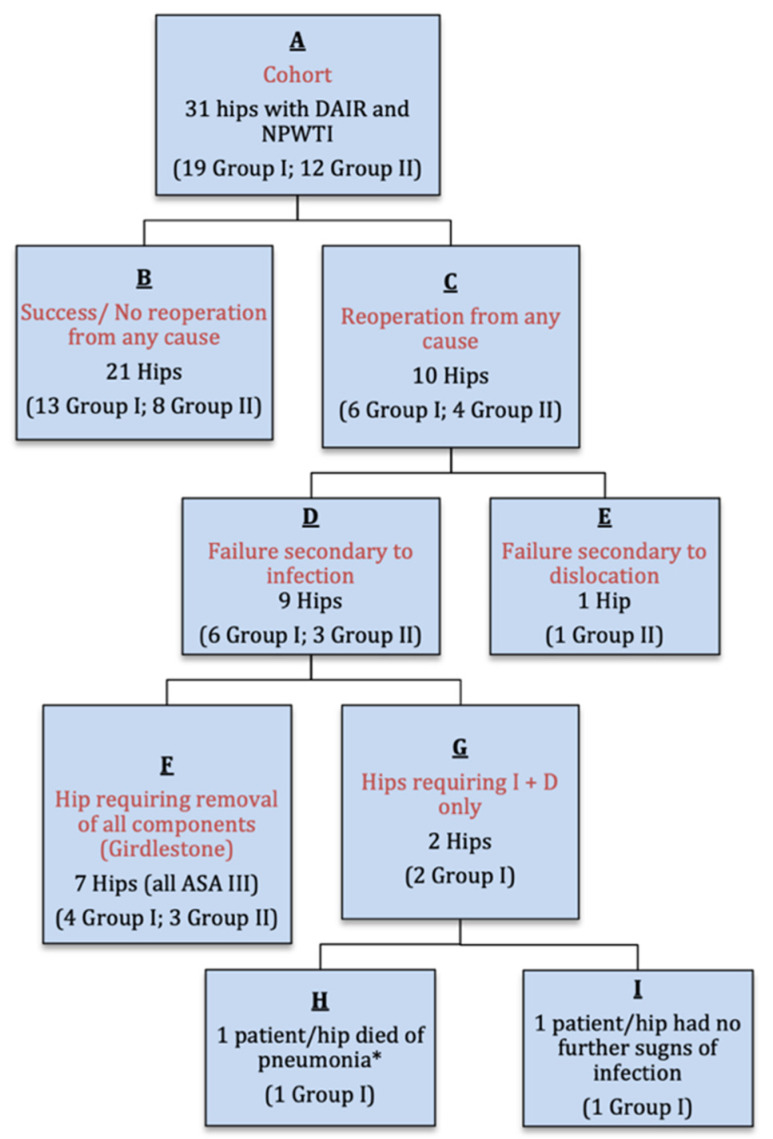
Flow chart (I + D = Irrigation and Debridement; * This patient in Group 1 was ASA Grade III and died 24 days post DAIR with MRSA and pneumonia and is considered a treatment failure).

Our cohort of 31 hips had 19 hips in group one and 12 in group two. Ten of these 31 hips required further surgery following the completion of our treatment protocol; nine were treated for infection and one for later dislocation. Of the nine hips treated for recurrent infection, seven required explantation (Girdlestone) and two required I and D only. One of the two patients treated with an additional I and D procedure died several days postoperatively as a result of pneumonia and sepsis. This was our only patient with an MRSA joint infection. This patient was considered a treatment failure (see Figure 1).

### 3.2. Outcome of Groups One and Two by Endpoint

The outcome was assessed for the following three study endpoints: elimination of infection with no further surgery for infection, implant survival from infection and implant survival from all causes, and were 71%, 74.2% and 71%, respectively. The influence of duration from the index procedure to DAIR was assessed by looking at the outcome results by patient group for each of the three endpoints. Our numbers were small, but we found no significant difference between groups one and two for any of the three endpoints (see Table 2).

### 3.3. Kaplan–Maier Analysis of Infection Related Implant Survival

**Figure 2:**
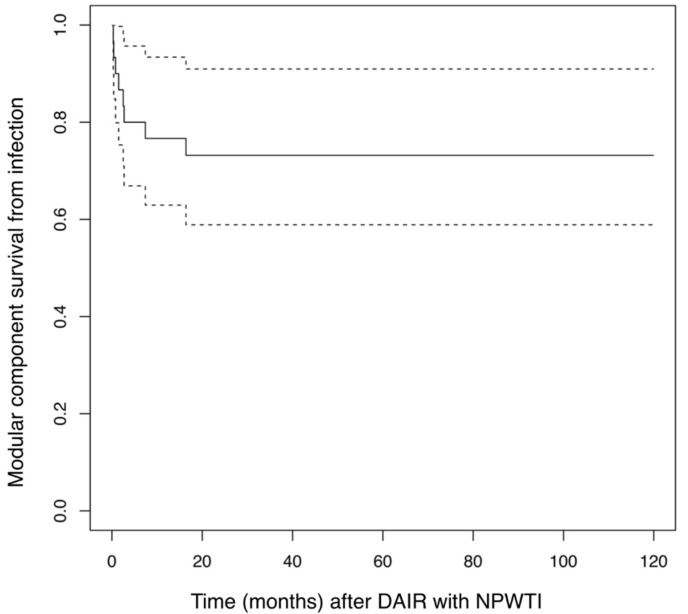
Kaplan–Maier curve showing infection-related implant survival after DAIR with NPWTI for an estimated 10 year follow up. The confidence intervals are represented by the dotted lines.

Kaplan–Maier analysis revealed a 60-month infection related implant survival of 73.2% (CI: 58.9–91.0%) with a corresponding re-revision rate secondary to infection of 26.8% (9.0–41.1%) after 5 years. The competing risk analysis was 26.7% (9.5–33.9%) for infection-related re-revision after 5 years (see Figure 2).

### 3.4. Organisms Cultured from Tissue Samples

Tissue samples taken during debridement had monomicrobial cultures in 19 hips, polymicrobial cultures in 8 hips and 4 hips were culture negative. Staphylococcus species, including one case of MRSA, were the most frequent pathogens and were cultured from 17 hips (63% of isolates). Streptococcus species were the next most frequent pathogen and were cultured from 7 hips (26% of isolates) (see Table 3).

Nine hips that required additional surgery for infection (seven Girdlestone and two I and D) had the following pathogens isolated from DAIR tissue samples: two hips were polymicrobial with Staphylococcus aureus and Coagulase Negative Staphylococcus, one hip was polymicrobial with Staphylococcus aureus and Streptococcus and there was one case each of monomicrobial E. Faecalis, Streptococcus, Staphylococcus aureus, E. cloacae, MRSA and one culture negative.

### 3.5. Postoperative Function Scores

Postoperative function, activity, pain and satisfaction scores were conducted at the final follow up. The HHS, WOMAC and satisfaction scores were in the ‘good’ range, pain was minimal, and the UCLA and Tegner Activity scores were low to moderate (see Table 4).

## 4. Discussion

Acute PJI constitutes 60–70% of all PJI cases [31]. The DAIR procedure has been the traditional treatment of choice for patients with a stable joint, sufficient soft tissue coverage and no sinus tract. However, DAIR remains controversial due to the great variability in reported success rates ranging from 16–100%. A 100% success has been reported in series with very small cohort numbers of 5–8 patients [13].

The terms ‘acute’ and ‘early’ have been used interchangeably for PJI to denote acute exogenous symptoms. The time frame from index procedure to acute symptom onset has been variously defined as up to 4 weeks [14,18,19,31,32], 6 weeks [17] or up to 90 days [10,20,33,34]. The term ‘acute hematogenous’ PJI has been used for acute symptoms occurring more than 3 months [10], 2 or more years [20] or any time [18,35] after index surgery in a previously well-functioning hip. Some authors required identification of a primary source or suspicion of bacteremia [18,36,37] and others did not [20,31]. Koyonos used the term ‘acute delayed’ for patients with acute onset and/or an identified source of infection occurring after postoperative day 28 [19]. We chose to exclude the term ‘acute hematogenous’ for our later onset cases, as we were unable to identify a primary source of infection in most cases. The lack of an identified primary source did not change the treatment protocol.

The length of time from index surgery to DAIR has had a variable effect on patient outcome, including a positive correlation between increased time and increased failure [38] or no significant difference before or after a 4-week interval [16,32,33,39]. Grammatopoulos et al. found a better outcome if the treatment occurred within 6 weeks of the index surgery, but a satisfactory outcome up to 13 weeks [17]. A comparison of our two patient groups (<6 weeks and >6 weeks) showed no significant difference.

However, the time interval between symptom onset and DAIR is generally considered to have great significance for the outcome [40]. The earlier the operative intervention, the greater the success [21,32,40,41,42]. Tsang et al., in their meta-analysis, found 20% greater success in studies with <7 days median time between symptom onset and debridement compared to those with >7-day interval [16].

Our cohort received surgical intervention within 24 h of acute symptom presentation, if medically stable. We utilized symptom presentation rather than symptom onset to minimize the possible errors of symptom misinterpretation and recall. We found no significant outcome difference between our two groups.

The successful treatment of acute PJI must address bacterial biofilm in addition to free-floating (planktonic) organisms. Biofilm develops after planktonic forms adhere to surfaces, multiply and secrete a film that is initially unstable but matures in 2–4 days to a more resistant form [16,25,43,44,45] that protects from antimicrobials and host immune responses [43,46]. Although biofilm is more difficult to culture than planktonic forms and may contribute to negative cultures [47], we believed that the attainment of negative cultures was our best indicator of infection elimination. The elimination of biofilm is a treatment challenge but is crucial since residual biofilm is a probable significant contributor to recurrent infection.

We achieved negative tissue cultures in all the hips prior to closure, but two hips later required additional I and D for a continued infection. We hypothesize that these hips had residual biofilm that yielded false negative tissue cultures; therefore, our negative tissue cultures were not a guarantee of biofilm/infection elimination. Our protocol of repeated debridement, NPWTI and polyhexanide instillation was designed to more effectively target biofilm and planktonic forms but was not effective in all cases, although polyhexanide is an effective anti-biofilm agent [48].

Focused antimicrobial therapy based on the antibiogram to target identified pathogens was administered throughout and continued for 6-8 weeks after surgical intervention. The traditional guidelines for antimicrobial therapy of acute PJI have been 3 months, but 8 weeks has been found to be equally affective [40,49,50]. We hypothesized that this protocol would reduce our need for later additional DAIR, I and D or chronic antibiotic therapy to treat recurrent infection, as used in some other studies (see Table 5) [17,21,31,32,41,51]. We also administered a battery of functional scales postoperatively and looked at failure from causes other than infection. At a mean follow up period of 4.3 years, infection elimination was achieved in 71% of the hips (22 hips) and implant survival from infection was achieved in 23 of 31 hips (74.2%).

A comparison between studies is difficult because of the differences in all aspects of the studies including cohort characteristics (hip only, hip and knee, PTHA only, PTHA and RTHA) [31,32,33,37,39,51,52] and treatment modalities, including antibiotic therapy [5,12]. Our protocol that involved repeat DAIR in most cases during one admission, makes comparisons difficult. In addition, there are relatively few studies that have both PTHA and RTHA patients with moderate or long follow up times and no chronic antibiotic suppressive therapy.

The definitions of success differed between the studies, but most authors considered success as infection elimination/control with implant retention and allowed for later additional DAIR/I and D procedures [17,21,31,32,53,54]. This definition corresponded to our endpoint of infection-related implant survival (retention) that included later I and D (see Table 5). Studies with no later additional DAIR/I and D [18,55] approximated our endpoint of infection elimination with no further surgery for infection and are listed in Table 6.

Our outcomes are listed in Table 5 and Table 6 with the caveat that our protocol routinely utilized multiple DAIR for most hips during one admission in contrast to other studies.

Multiple factors affect outcomes, including patient co-morbidities, intra-operative variables, antibiotic protocol and the organism (s) involved. An increased BMI, high ASA scores and compromised host immunity have been associated with increased failure [38,42,56]. Our cohort had a mean BMI of 27.3 (range 18.9–39.3) kg/m^2^ and all but one patient were in ASA groups II–IV.

Other factors include the exchange of modular components that is generally considered to be of great importance for success. Koyonos performed DAIR without modular component exchange on 138 knee and hip joints and achieved a 35% success rate [19]. Tsang et al. found a 13.3% improved DAIR outcome in studies that utilized modular exchange compared to those that did not [16]. Modular exchange was thought to allow improved access for debridement [40,42] and up to twice the probability of long-term remission [52]. Veltman et al. stated that the Netherlands Orthopedic Association concurred with the recommendation of the International Consensus Meeting of 2014, which stated that all modular components should be exchanged during DAIR [15,57].

The effect on the outcome of multiple I and D procedures is unclear, particularly if there is a diminished host response or a more virulent organism. [40,58,59,60,61,62,63,64]. An International Consensus in 2013 recommended the consideration of implant removal after failure of one I and D but more I and D’s can be performed with some protocols [15,41]. Some have suggested that a prior failed I + D procedure may reduce the chance of a later successful two-stage implantation resulting in a 34% failure rate, but this has been refuted in more recent US studies [42,65,66,67,68]. In our series, most patients had multiple debridements in association with NPWTI, as dictated by the presence of residual infection and outlined in the treatment protocol described by Lehner et al. [9,69].

As in other series [18,21], the Staphylococcus species was our most frequent pathogen and was present in 63% of isolates. Staphylococcus aureus, including one isolate of MRSA, was cultured in 26% and coagulase negative Staphylococcus in 37% of cases. Streptococcus and Enterococcus were isolated in 26% and 15% of cases, respectively. All are efficient producers of biofilm. Staphylococci are thought to produce 50% of all the biofilm on medical devices [34,43,44,70,71] and have been implicated in poorer DAIR outcomes [19,40,49,51,62,72]. Biofilm can also be polymicrobial [16,25,45]. A 2013 multi-center study [49] documented that MRSA was not associated with a worse outcome, although our only patient with MRSA infection succumbed with pneumonia.

Documentation of functional outcomes and postoperative complications is sparse. Westberg reported a mean HHS of 86 in 38 patients—all with Primary THA as index surgery [32]. At the final follow up, our patients reported a mean HHS of 81.9 (good = 80–90), median pain score of 1, WOMAC score of 31.7 (mild/moderate functional disability) and mean UCLA and Tegner activity score of 5 and 3, respectively (midrange).

We had one postoperative dislocation (3.3%). Other series have reported 21 [32] and 14% [17] dislocations, respectively.

## 5. Limitations

This study was based on a retrospective chart review with the attendant risks of incomplete documentation.

Our heterogeneous patient collective was small, and we lacked a control group treated without NPWTI.

The cohort was mostly ASA groups II and III, with serious co-morbidities that may have excluded them from consideration for DAIR in studies with more stringent selection criteria.

Multiple surgeons performed the procedures and variations in technique cannot be excluded, although the protocol and procedures were the same throughout.

The small size of our cohort precluded the assessment of variables that could potentially affect the outcome, such as repeated debridement/instillation or the nature of the offending pathogen(s).

Rather than the customary timeline of symptom onset, we utilized the more objective criterion of symptom presentation to medical attention, with the risk of the under-estimation of symptom duration.

## 6. Conclusions

The results for both our patient groups showed no significant difference and were in line with those of prior studies, despite the differences resulting from heterogeneity in all aspects of the studies. We also achieved a good HHS score and had only one postoperative dislocation.

Many studies that reported similar or better results employed different definitions of success, including later additional DAIR/I and D procedures [17,21,31,32,41,51] or the use of ancillary long term antibiotic therapy [17,31,51] that we considered to be a postoperative complication. Some also had more stringent inclusion criteria, such as PTHA cases only [31,32] (see Table 5).

Our treatment protocol was designed to more completely eliminate bacterial biofilm. Our treatment protocol was designed to more effectively target biofilm. We hypothesized that by eliminating biofilm as documented by the achievement of negative tissue cultures, we would markedly reduce or eliminate the need for later additional DAIR/I and D. However, we were unsuccessful in two cases. Therefore, the attainment of negative tissue cultures did not guarantee the elimination of biofilm/infection, and our results showed no advantage over other studies in regard to infection control.

The comparative utility of our protocol is inconclusive because this methodology has been rarely used to date and ours is a single study with no control group and a small cohort. Only further employment could indicate its effectiveness. The potential advantage of a possibly reduced bacterial biofilm and a reduced number of later additional DAIR/I and D procedures must be weighed against an increased number of procedures per patient with an associated lengthy and arduous hospitalization that incurs increased financial expenditure.

## Figures and Tables

**Table 1:** Reason for Index Operation.

Procedure	Indication	Number (n)	
Primary Index Operation	Idiopathic Osteoarthritis	15	hips
	Osteoarthritis secondary to hip dysplasia	5	hips
	Osteoarthritis secondary to trauma	1	hip
Revision Index Operation	Liner wear	1	hip
	Dislocation	3	hips
	Metallosis	1	hip
	Loosening of femoral shaft	1	hip
	Loosening of acetabular cup	1	hip
	Pseudarthrosis after periprosthetic fracture	1	hip
	THA Reimplantation after Girdlestone	2	hips

**Table 2:** Shows treatment outcome by group after a mean follow up of 4.3 ± 3.7 years.

Outcome	Group I (19 Hips)	Group II (12 Hips)	Total (31 Hips)
Infection eliminated with no further surgery for infection (B + E)	13 (68%)	9 (75%)	22 (71.0%)
Infection related implant survival (B + E + I)	14 (73.6%)	9 (75%)	23 (74.2%)
Implant survival from all causes (B + I)	14 (73.6%)	8 (66.7%)	22 (71.0%)

**Table 3:** Organisms cultured in entire patient cohort.

Organism(s) Isolated	Total # Isolated	Monomicrobial	Polymicrobial
Staph. Aureus	6	3	3
MRSA	1	1	0
Coagulase Negative Staphylococcus Epidermidis	5	2	3
Coagulase Negative Staphylococcus Hominis	1	1	0
Coagulase Negative Staphylococcus lugdunesis	2	1	1
Coagulase Negative Unspecified	2	0	2
Streptococcus non-Haemolytic	6	5	1
Haemolytic Group G	1	1	0
Enterococcus	3	2	1
Enterobacter	2	1	1
Escherichia coli	3	1	2
Miscellaneous/other	4	0	4
Culture negative	4		

**Table 4:** Post-operative Function and Pain Scores.

Post-Operative Scores	Mean ± Standard Deviation	Median	Range	Interquartile Range
Harris Hip Score	81.1 ± 11.8		52–95	73.5–91.5
WOMAC	33.3 ± 20.1		2.1–67.7	12.0–46.4
Pain (VAS) (0–10)		1	0–5	0–2
Satisfaction (1–5)		2	1–5	2–3.5
Activity (UCLA) (1–10 Scale)		5	2–8	3–6
Activity (Tegner) (0–10 scale)		3	1–4	2–3

**Table 5:** Studies showing infection elimination/control with additional DAIR and I and D.

Author	Year	# of Acute PJI of Hip	Follow Up Months	Primary, *p*. Revision, R	Success	Modular Exchange	Long-Term Antibiotics
Crockarel	1998	23	75	*p* = 20; R = 3	26%	0%	No
Westberg	2012	38	48	*p* = 38	71%	100%	No
Sukeik	2012	26	79	*p* = 16, R = 10	77%	100%	No
Triantafyllopoulos	2015	60	59	*p* = 38, R = 22	79%	100%	No
Grammatopoulos	2017	122	84	*p* = 82, R = 40	85%	53%	Yes
Bryan	2017	90	72	*p* = 90	83%	70%	Yes
Beckmann	2021	31	52	*p* = 21, R = 10	74.2%	100%	No

**Table 6:** Studies showing elimination/control of infection with no further DAIR/I and D for Infection.

Author	Year	# of Acute PJI of Hip	Follow Up Months	Primary, *p*. Revision, R	Success	Modular Exchange
Tsukayama	1996	41	45	Unknown	68%	100%
Klouche	2011	12	40	*p* = 6, R = 6	75%	40%
Beckmann	2021	31	52	*p* = 21, R = 10	71%	200%

## Data Availability

The data that support the findings of this study are available on request from the corresponding author.
